# Are monkeys intuitive Aristotelians? Associations between target size and vertical target position in long-tailed macaques

**DOI:** 10.1098/rsos.170889

**Published:** 2018-04-11

**Authors:** Stefanie Keupp, Natàlia Barbarroja, Sascha Topolinski, Julia Fischer

**Affiliations:** 1 Cognitive Ethology, Deutsches Primatenzentrum GmbH, Kellnerweg 4, 37077 Göttingen, Germany; 2 Fundació Universitat de Girona: Innovació i Formació, Girona, Spain; 3 Department of Psychology, Social and Economic Cognition Cologne, University of Cologne, Richard-Strauss-Straße 2, 50931 Köln, Germany

**Keywords:** spatial compatibility effects, monkeys, cognitive effort, size discrimination

## Abstract

Different hypotheses have been put forward to explain the interaction between size perception and spatial position. To explore the evolutionary roots of these phenomena, we tested long-tailed macaques' performance in a two-choice discrimination task on a touchscreen and contrasted two hypotheses. First, a hierarchy association in which large objects are associated with top positions, due to a link between power, dominance and importance with top position. Second, a naive Aristotelian association in which large objects are associated with bottom positions, due to the experience that larger objects are heavier and thus more likely to be found at the bottom. Irrespective of training regime (positively reinforcing the small (Touch-Small) or large (Touch-Large) stimulus), the monkeys had a bias to touch the bottom compared to the top location. Individuals in the Touch-Small group took significantly longer to acquire the task, but subsequently made fewer mistakes. When presented with two stimuli of equal medium size, the Touch-Large group had a clear bias to touch the lower stimulus, while the Touch-Small group touched both locations at equal rates. Our findings point to an innate bias towards larger stimuli and a natural preference for the lower position, while the extent of interaction between size and position depends on executive control requirements of a task.

## 1. Introduction

Categorization of stimuli can be modulated by their spatial position. For example, people who were required to perform a parity judgement task on numbers presented at different locations on a PC screen categorized higher numbers faster when they were presented at the top compared to the bottom of the screen, while lower numbers were categorized faster when they were presented at the bottom compared to the top of the screen [1]. This effect is known as spatial-numerical association of response codes (the so-called vertical ‘SNARC effect’) and is one type of spatial compatibility effect (for an overview, see [2]). These findings are not easily explained by skills or processes that depend on formal education, experience or abstract thinking: although cultural habits, such as writing direction, do contribute to the horizontal SNARC effect [3,4], a number of studies have shown spontaneous mapping of numbers or quantities to horizontal space in preverbal human infants [5–8] and some animal species (e.g. rhesus macaques [9] and pigeons [10]). This suggests that such effects do not depend on factors of enculturation alone. For example, Bulf *et al.* [5] recently found that 9-month-old infants' visual attention to left or right could be modulated by presenting high or low numerical cues at a centre position. This left-to-right bias has also been shown in 4-year-olds, an age in which most children cannot yet read or write [7]. Surprisingly, such spatial-numerical associations are not human-specific. For example, Rugani *et al.* [11] recently found number-space mapping in young domestic chicks who spontaneously associated smaller numbers with left and larger numbers with right. Rhesus macaques showed spatially oriented number mapping when they had to transfer a learned response to a new stimulus array [9]: after being trained to select the fourth position from the bottom of a five-element vertical array, they preferentially chose the fourth stimulus from the left (rather than from the right or at random) in a horizontally presented array.

Regarding the neuronal and biological basis for these findings, several lines of research (neuronal imaging and intervention, behavioural, neuropsychological) indicate a close connection between spatial and numerical magnitude processing and stress the crucial role of parietal associative cortical regions, in which these processes share some neuronal circuitry (e.g. [2,12–14]). Along these lines, Drucker & Brannon [15] as well as Adachi [16] suggested that this might indicate a reuse of systems that are already in place, such as mechanisms for spatial attention, and perhaps even enables the understanding of abstract concepts that cannot be perceived via sensory organs. Such links between sensorimotor components and action in cognition support the notion of *embodiment* [17,18], *grounded cognition* [19], *common coding* [20] or mental representations and cognition as *perceptual symbols* [19,21]; such theories hold, broadly speaking, that perception, action and cognition are directly linked and not modular as earlier cognitive accounts assumed (e.g. [22]).

Associative links, particularly for the spatial domain, have also been demonstrated for representations other than numbers and quantities. For example, the concept of power and status in the social hierarchy has been shown to be associated with the spatial dimension of verticality, both in humans [23,24] and in chimpanzees [25]. The latter study found that chimpanzees spontaneously mapped higher-ranking individuals to the upper position on a screen. More specifically, in a match-to-sample task, chimpanzees were quicker to identify the matching picture of higher-ranking group members when it was presented on the upper location on a touchscreen, indicating spontaneous mapping of rank to vertical location. Thus, at least for humans and chimpanzees, there might be a generic association between ‘more’ and top and ‘less’ with bottom, potentially encompassing other domains in addition to number quantity. While we are not aware of a similar experiment with long-tailed macaques (*Macaca fascicularis*), the species studied in this paper, observations of locomotive behaviour of long-tailed macaques in the wild indicate that males—who are higher ranking than females—usually occupy higher vertical positions in trees unless they are following females during the mating season, in which case they can be found at lower heights ([26], C. Girard-Buttoz 2018, personal communication). Hence, it is possible that long-tailed macaques spontaneously associate upper location with higher position in the hierarchy. In humans, the association of rank and vertical position is also evidenced in many cultural set-ups and icons, such as displays of social hierarchies, or the use of pedestals for leaders and winners [27]. A recent study [28] provided strong evidence for this notion. In this paradigm, human participants engaged in a comparative judgement task and evaluated the physical size of two stimuli, for instance, the visual size of two letters. Crucially, these stimuli were presented randomly at the top or bottom location of the PC screen. Participants made fewer errors when the stimulus with the higher value appeared at the top and the stimulus with the lower value at the bottom; conversely, they made more errors when the stimulus with the higher value appeared at the bottom and the stimulus with the lower value at the top.

If large numbers and quantities are associated with vertical top positions (as in the vertical SNARC effect), and powerful social agents are associated with vertical top positions, it is likely that the mere abstract concept of *more* is also associated with top positions (and conversely for small/powerless/less ∼ bottom). The pioneering embodiment theorist Lakoff put this idea as follows: ‘Whenever we add more of a substance—say, water to a glass—the level goes up. When we add more objects to a pile, the level rises. Remove objects from the pile or water from the glass, the level goes down. The correlation is overwhelming: more correlates with up, but less correlates with down’ [29].

Particularly, when it comes to size as a judgemental dimension, however, one might also argue for a reversed, ecologically equally relevant association: larger objects are usually heavier than smaller objects of the same kind and thus are more likely to be found closer to the ground (for the psychological embodiment of the concept of weight, see e.g. [30]). For example, non-human animals experience that a large fruit hanging from a branch usually causes more bending towards the ground, while a small fruit causes less bending of the branch and thus hangs higher compared to the large fruit. This notion equals naive early physical theories on weight, most famously Aristotle's view on object movements [31]. In his classic work ‘De Caelo’ (‘On the heavens’, [32]), Aristoteles laid out his intuitive understanding of gravity. He claimed that every object strives to its natural place, with heavy, earthy objects striving to lower places and light, aerial objects striving to the top. Specifically, his notion is that the more ‘earth’, the more solid substance an object has, the lower it goes. Since the amount of substance correlates with the size of an object, we call the association larger-bottom and smaller-top an intuitive Aristotelian view. The present experiment contrasted the ‘hierarchy’ and ‘Aristotelian’ hypotheses regarding spontaneous vertical associations of stimuli.

In this study, we explored the link between vertical arrangements of two stimuli (top versus bottom) and judgements of the size of the target stimuli during a simple discrimination task in long-tailed macaques. Specifically, we investigated whether the mere (top versus bottom) location of a target stimulus (in this case, the smaller or larger of two shapes) would activate the semantic concept of ‘more’ and ‘less’ quantity in the current size discrimination task. Monkeys and great apes have been shown to discriminate quantities and sizes [33–36], but it has so far not been investigated whether the processing of and response to stimuli that differ in physical features might be influenced by their spatial location. In addition, while it has been shown that some animals (rhesus macaques [9], domestic chicken [11], chimpanzees [16]) express horizontal spatial-numerical mappings indicating a mental number/magnitude line, we are not aware of similar findings of spontaneous magnitude-space associations in the vertical dimension. If monkeys maintain a ‘hierarchical’ representation, like humans (see [28]) and chimpanzees [25], and thereby a large-top and small-bottom association, their responses of size discrimination would be quicker and more accurate when the larger (smaller) stimulus is presented on the top (bottom) position. In turn, this would also imply that our ‘embodied’ cultural representations might be rooted in an evolutionary more ancient bias. If, on the other hand, the monkeys maintain a rather ‘Aristotelian’ representation involving simple gravitational-weight relations, we expect the opposite pattern: responses will be quicker and more accurate when the larger (smaller) of two shapes is presented at the bottom (top). This would imply that at some point in evolutionary history such pragmatic associations were complemented with or replaced by a hierarchical representation. We have summarized these predictions in table 1.

**Table 1.** Predictions for Aristotelian and hierarchy hypothesis regarding hypothesis-congruent and hypothesis-incongruent displays in the different-size test and preferred touch location in the same-size test.

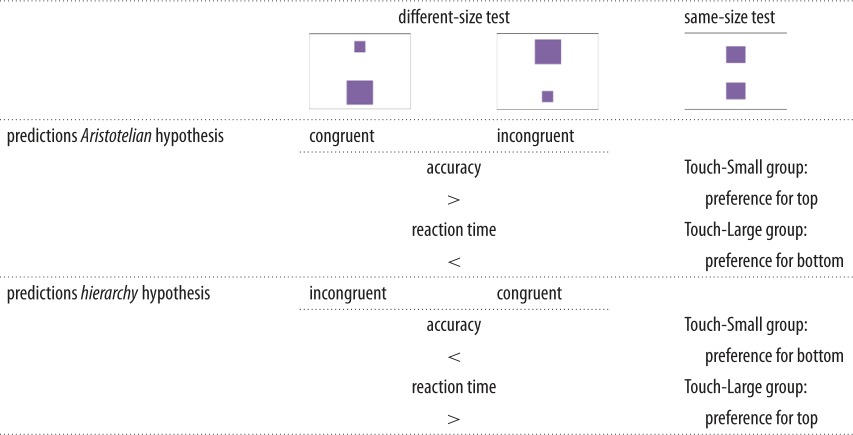

## 2. Methods

### 2.1. Subjects

A total of 12 long-tailed macaques (five females, seven males; age range 1–8 years) participated in this study (table 2). The monkeys are part of a larger research project on comparative cognition and have been involved, for instance, in studies on physical and social cognitive abilities [33] and quantity discrimination [34]. The monkeys are housed in a social group of 38 individuals at the German Primate Center in Göttingen and have access to indoor (49 m^2^) and outdoor enclosures (141 m^2^), which are equipped with trunks, branches, fire hoses, wooden platforms and other enriching objects. During the summer months, they have access to water basins, which they use extensively for playing and manipulating enrichment objects or food items in the water. Monkeys were tested individually and participation was voluntary, i.e. dependent on the monkeys' willingness to enter the testing compartment. One female stopped participating due to giving birth and her data were only used for the analysis of Training Stage 1. Some subjects had participated in previous touchscreen tasks unrelated to the present study [37]. Water was always available ad libitum, and subjects were not food deprived for testing. Long-tailed macaques in the wild live in female bonded multi-male/multi-female groups with a despotic social style [38]. Groups are organized along matrilineal social hierarchies, while males disperse. They are opportunistic omnivores and their diet consists of fruits, leaves, flowers, roots, insects, occasionally catching small vertebrates, bird eggs and crabs in some groups.

**Table 2.** Test subjects.

name	sex	date of birth	test group
Isaak	m	10 Apr 2011	Touch-Large
Lord	m	4 Feb 2014	Touch-Large
Mars	m	17 Jan 2014	Touch-Large
Max	m	1 Feb 2013	Touch-Large
Milka	f	29 Dec 2014	Touch-Large
Sophie	f	3 Apr 2009	Touch-Large
Ilja	m	29 Dec 2012	Touch-Small
Selina	f	20 May 2008	Touch-Small
Linus	m	16 Jan 2013	Touch-Small
Maja	f	17 Oct 2007	Touch-Small
Mila	f	7 Apr 2012	Touch-Small
Snickers	m	12 Jan 2014	Touch-Small

### 2.2. Set-up

**Figure 1.**
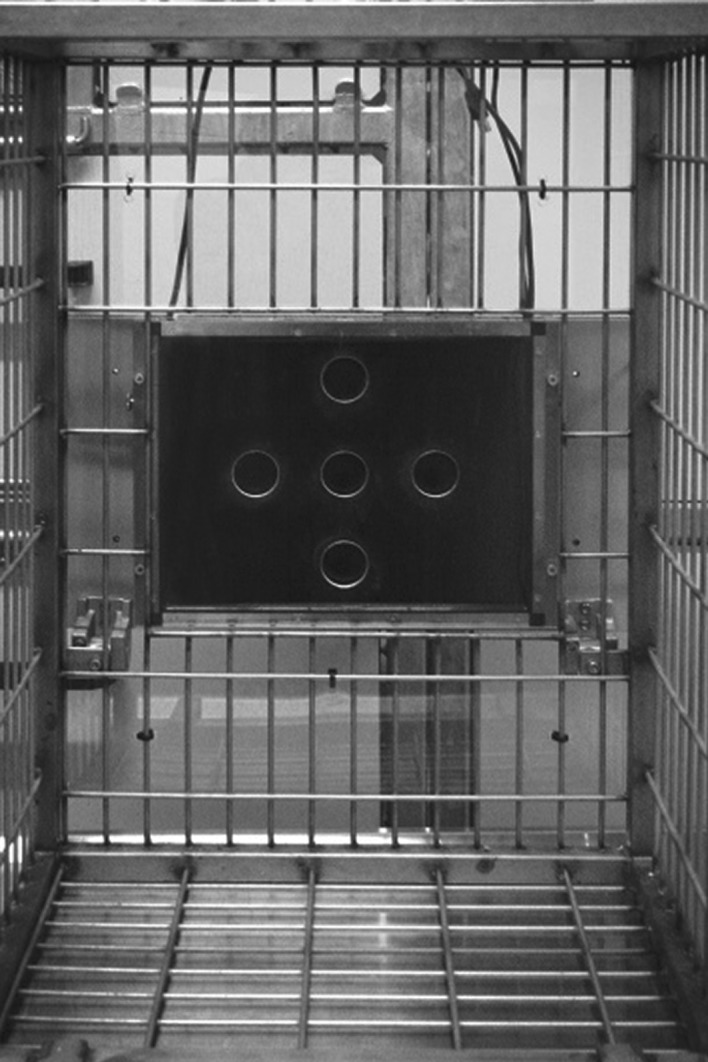
Touchscreen set-up from monkey perspective. The centres of the holes were positioned at 24, 34 and 44 cm height, respectively.

Data collection took place in a designated testing area (2.6 m × 2.25 m × 1.25 m; height, width, depth) directly adjacent to the indoor enclosure. The set-up consisted of a 15-inch touch-sensitive monitor (Elo Touch Solutions), which was attached vertically in front of a window in the cage. A Plexiglas panel with five holes of ∅ 3.5 cm was installed in front of the monitor (figure 1). Holes corresponded to the positions on which stimuli could appear on the screen and we found that the panel helped the monkeys to touch more precisely and with only one hand at a time. *MWorks* software (MWorks v. 0.6, c1863e7, 2002–2015 The MWorks Project) on a MacBook was used for stimulus presentation and data collection. Response-contingent feedback sounds were played via an external loudspeaker attached to the computer. Reward delivery was performed by the experimenter: monkeys received a raisin for each correct answer. Test sessions were videotaped but data analysis was conducted with data collected with *MWorks*.

### 2.3. Procedure

Training and testing phases consisted of four consecutive parts: (i) familiarization, (ii) horizontal training, (iii) different-size test and (iv) same-size test. During the familiarization, subjects were habituated to the experimental set-up and learned to touch a shape presented on the touchscreen. In the training stages, a large and a small stimulus of the same category (e.g. squares) were arranged horizontally (left and right position). Half of the subjects had to touch the smaller and the other half had to touch the larger of the two stimuli (throughout the article, we refer to the different reward category groups as ‘Touch-Small group’ and ‘Touch-Large group’, respectively). Individuals were randomly assigned to one of these groups. In the different-size test, a large and a small stimulus of the same category were presented in a vertical arrangement (top and bottom position). We predicted that if monkeys maintain an Aristotelian association, their responses of size discrimination would be quicker and more accurate when the larger stimulus is presented on the bottom position—and vice versa for the hierarchy association. The same-size test differed from the different-size test in that both stimuli were of identical medium size. If individuals who are looking for the large stimulus spontaneously prefer to touch the bottom (top) position this would speak in favour of the Aristotelian (hierarchy) association. For Touch-Small group individuals, the opposite holds: a preference to touch the top (bottom) position would speak in favour of the Aristotelian (hierarchy) hypothesis. A subject could take part in up to three sessions per day, except for the same-size test phase, in which they only participated in two sessions per day due to the higher number of trials per session (see below).

#### 2.3.1. Stimuli

Six symmetrical shapes were used as stimuli (see electronic supplementary material, figure S1). Shapes were created by PowerPoint in opaque purple colour and 200 × 200 pixel size. The standard size was 4 cm × 4 cm; sizes used in the experiments are given below. Importantly, small and large stimuli had touch-responsive areas of equal size surrounding them to ensure that the likelihood to hit the small shape when aiming for it was equally high as the likelihood to hit the large shape.

#### 2.3.2. Familiarization

All but one subject had participated in a touchscreen study before, in which they had to discriminate pictures of men and women ([37]; S. Keupp *et al.* 2015/2016, unpublished data). General set-up and stimuli were different from the former study and, therefore, all monkeys first received a general familiarization and training, in which they learned that touching the presented stimuli results in receiving a food reward. Importantly, there was no specific objective criterion to ‘pass’ this phase, but rather our aim was to ensure that the monkeys felt comfortable with the new set-up and understood what was required in order to receive a reward (i.e. to interact with the touchscreen). This was decided upon personal assessment by the experimenter, who had worked with this group of monkeys before. For this ‘training to touch’, all of the shapes were used, however monkeys saw only one stimulus at a time. Presentation of stimuli and position was randomized across the five Plexiglas hole positions. Upon a successful touch, the stimulus disappeared, a sound played (frequency = 300 Hz, duration = 100 ms; later referred to as ‘correct’ sound) and one raisin was given as reward by the experimenter. The experimenter stood in front of the test cage and handed the reward to the monkeys at central position below the touchscreen. If a touch was outside the responsive area of the stimulus, the display did not change. To make their touch more accurate, subjects first had to touch shapes of a size of 6.4 cm × 6.4 cm (i.e. 60% bigger than the designated standard size). When their touch was accurate enough stimulus size was decreased to 4.6 cm × 4.6 cm (i.e. 15% bigger than the standard size and equivalent to a medium size between the large and small test stimuli). As soon as a subject was reliably touching the stimuli on the screen, they proceeded to the actual training phase.

#### 2.3.3. Horizontal training

During the training phase, subjects had to learn to touch the smaller or larger of two horizontally arranged stimuli, depending on their assigned reward category. A training session consisted of 24 trials, which the subject could initiate by touching a start sign at the centre of the screen. The start sign then disappeared and two shapes were presented on the left and right positions. Each stimulus appeared equally often on both sides and presentation order was randomized. Within a trial, only shapes of the same category were presented (e.g. small square and large square, but not small square and large diamond). The size difference between stimuli was 30% (large: 5.2 cm × 5.2 cm, small: 4 cm × 4 cm) and stimulus centres were 10 cm apart (to prevent an overlap of responsive areas). Stimuli remained on the screen until a response was given. If a subject successfully touched the correct stimulus the ‘correct sound’ was played, the stimuli disappeared and the subject was rewarded with a raisin from the hand of the experimenter. After 3 s, the start sign appeared to signal the beginning of the next trial. If a subject did not take the reward within these 3 s, the raisin was removed. If the subject touched the incorrect stimulus, the ‘wrong’ sound (frequency = 150 Hz, duration = 300 ms) was played and a red (255,0,0 RGB) full screen appeared for 3 s.

The horizontal training comprised three training stages, which only differed in the number of presented shape categories (stage 1: only square shape, stage 2: square and diamond, stage 3: square, diamond, hexagon, cross, vertical rectangle, horizontal rectangle; see also electronic supplementary material, figure S1). Once the subjects reached the success criterion (18 of 24 correct trials in two consecutive sessions), they proceeded to the next stage. The same criterion applied to all training stages. We chose this success criterion of 75% to ensure that individuals had understood the task well but were not performing at ceiling, in order to allow for potential spatial-bias effects to manifest in the test. Every subject did at least 120 trials (five sessions of 24 trials) in each training phase, independent of whether they reached the criterion before. This number of trials was determined by a power analysis (including the aspects: individual differences, side bias, difficulty of task—i.e. rewarded size category, learning progress, a possible embodiment component—i.e. a size-induced side preference, and interaction of stimulus category and learning speed) and information about effect sizes from earlier studies on embodiment in non-human animals prior to the start of the training [33].

An additional training stage was added for those subjects who performed at ceiling in Stage 3. A ceiling effect was defined as being correct in 22 or more trials on average, in the five sessions. In this case, we reduced the size difference between the shapes to 15% (large: 4.6 cm × 4.6 cm, small: 4 cm × 4 cm) and presented the experiment until they reached the criterion. None of the subjects performed at ceiling in the additional stage. Once the monkeys performed reliably at 75%, the test phase began. Note that there was no training to adjust to the new stimuli locations because spontaneous rather than learned responses to the vertical location of small and large stimuli were of crucial interest.

#### 2.3.4. Different-size test

The different-size test only differed from horizontal training stage 3 with respect to stimulus locations: the two stimuli were presented at the top and bottom location, respectively. All six shape categories were presented and stimulus size was chosen according to the size difference reached in training stage 3. Three subjects of the Touch-Large group were tested with the original size difference of 30% and the three other subjects of this group with the size difference of 15%. All Touch-Small group individuals were tested with 30% size difference as none of them had performed at ceiling in the horizontal training. Shape category and position were randomized within a session with each shape category appearing four times in total. As in the horizontal training, both stimuli were 10 cm apart. Each subject received 120 trials (five sessions of 24 trials each) and then proceeded to the *same-size* test, irrespective of their performance level.

#### 2.3.5. Same-size test

Two vertically arranged stimuli of identical medium size were presented (4.6 cm × 4.6 cm, i.e. between the size of the original small and large stimuli). A test session consisted of 48 trials: trials 1–24 were a repetition of different-size test trials (conditional rewarding according to touch-size category) and trials 25–48 were same-size trials (unconditional rewarding). All six shapes were presented and each subject received 120 same-size trials (five sessions of 24 same-size trials each). We chose to remind the monkeys of their respective reward contingencies because not all individuals could be tested every day and we wanted to ensure that larger gaps between sessions did not interfere with their memory of the task.

### 2.4. Coding and analysis

We were interested in response latencies and touch locations depending on reward category (Touch-Large or Touch-Small group) and stimulus position. *MWorks* registered and automatically logged every touch on the screen during an active session. Latency to touch a stimulus was calculated from the time of touching the start symbol in each trial. We analysed each training and test phase separately. Analyses were performed with R statistical computing environment using functions from the lme4 package [39].

Our main focus was on the two vertical test conditions. For the analyses of accuracy in the different-size test, we assessed the influence of the fixed factors *touch-size group* (large or small), *location of the target* (top or bottom) and their interaction on the probability to correctly touch the target stimulus. We included *session* as an additional potential confounding factor and *subject ID* as random factor. For the analyses of touch position in the same-size test, we assessed the influence of *touch-size group* on the probability to touch the bottom location. Also here, we included *session* as a potential confounding factor and *Subject ID* as random factor. Analyses of response latencies followed the same rationale.

We additionally analysed response patterns in the horizontal training stages because it appeared that individuals of the Touch-Small group had a harder time to learn the task than individuals of the Touch-Large group. Analysis of the horizontal training followed the same approach as for the different-size test.

For all analyses, we compared the null model with the model containing the variables of interest (using likelihood ratio tests with the *anova* function) to determine if the data is better explained by the latter. We provide *conditional R*^2^ effect sizes for those full models which were significantly different from their respective null models (using the function *r.squaredGLMM* of the package MuMin [40]). We only included trials with response latencies up to 3 s because longer reaction times rather reflected inattentiveness of the subject (due to fights in the group or other disturbances) than an indication for increased processing time (of a total of 10 470 trials 97 were excluded).

## 3. Results

### 3.1. Horizontal training

#### 3.1.1. Stage 1 (square only)

##### 3.1.1.1. Descriptive analysis

Touch-Small group individuals needed on average more sessions to reach the learning criterion than Touch-Large group individuals: *m*_(small)_ = 14.4 sessions (range = 8–16), *m*_(large)_ = 6.1 sessions (range 2–14). One subject of the Touch-Small group stopped participating after session 8 without reaching the criterion. To assess the effect of response category on accuracy, further analyses were confined to the first five sessions per individual, as this was the originally determined minimum number of sessions per training stage. Note, however, that for two subjects of the Touch-Large group the stage 1 training was ended wrongly (due to experimenter mistake) after they reached criterion after two sessions. Since these individuals did not express particular difficulties in training stage 2, we do not consider this a reason for exclusion (see electronic supplementary material, table S1 and figure S2 for further details of the monkeys' response behaviour). A total of 1281 observations of 12 individuals are included in this dataset (16 trials were excluded due to latencies greater than 3 s).

##### 3.1.1.2. Statistical analysis

The full model (containing touch-size group, target position and their interaction; see electronic supplementary material, table S2) was significantly different from the null model (*χ*^2^ = 164.61, d.f. = 3, *p* < 0.001, *conditional R*^2^ = 0.309). We found a significant interaction of touch-size group and target position (*χ*^2^ = 96.46, d.f. = 1, *p* < 0.001), indicating that target position influenced the individuals of the two groups differently. While individuals of the Touch-Small group touched both positions equally often, thus showing at-chance performance, the Touch-Large group individuals touched the target more often correctly especially when it was presented on the left side. We also assessed if reaction times differed depending on touch-size group and target position. For correct trials, response latencies were shorter on the left compared to the right position (*χ*^2^ = 7.527, d.f. = 2, *p* = 0.023, see also electronic supplementary material, table S3). For incorrect trials, the full model did not differ significantly from the null model (*χ*^2^ = 1.81, d.f. = 3, *p* = 0.613).

#### 3.1.2. Stage 2 (two shapes)

##### 3.1.2.1. Descriptive analysis

In general, subjects were slightly quicker in reaching the success criterion in stage 2 compared with stage 1: *m*_(small)_ = 6.2 sessions (range = 5–8), *m*_(large)_ = 5.2 sessions (range 5–6). Five Touch-Large group individuals did five sessions and one individual needed an additional session to pass. For the Touch-Small group, two individuals passed after five sessions, and the others needed six, seven and eight sessions, respectively (see also electronic supplementary material, figure S4). Electronic supplementary material, table S4 gives an overview of response behaviours by stimulus arrangement and touch-size group for the first five sessions; 1313 observations of 11 individuals are included in this dataset (nine trials were excluded due to latencies greater than 3 s).

##### 3.1.2.2. Statistical analysis

The full model (containing touch-size group, target position and their interaction; see electronic supplementary material, table S5) was significantly different from the null model (*χ*^2^ = 23.593, d.f. = 3, *p* < 0.001, *conditional R^2^* = 0.122). We found a significant interaction of touch-size group and target position (*χ*^2^ = 13.405, d.f. = 1, *p* < 0.001), indicating that target position influenced the individuals of the two groups differently. We also assessed if reaction times differed depending on touch-size group and target position. For both correct and incorrect trials, the full model did not differ significantly from the null model (correct trials: *χ*^2^ = 3.942, d.f. = 3, *p* = 0.268; incorrect trials: *χ*^2^ = 1.199, d.f. = 3, *p* = 0.753).

#### 3.1.3. Stage 3 (six shapes)

##### 3.1.3.1. Descriptive analysis

All individuals of the Touch-Large group passed the learning criterion within the first five sessions (see electronic supplementary material, figure S4). Only two individuals of the Touch-Small group managed to pass within five sessions, the others needed six, 10 and 16 sessions, respectively: *m*_(small)_ = 8.4 sessions (range = 5–16), *m*_(large)_ = 5 sessions (range 5–5). Electronic supplementary material, table S6 gives an overview of response behaviours by stimulus arrangement and touch-size group for the first five sessions; 1303 observations of 11 individuals are included in this dataset (17 trials were excluded due to latencies greater than 3 s). As explained above, three individuals of the Touch-Large group performed at ceiling and, therefore, we increased the level of difficulty for them by decreasing the size difference between the small and the large stimulus. Two individuals reached training criterion with the more difficult task after five sessions and one individual needed 10 sessions.

##### 3.1.3.2. Statistical analysis

The full model (containing touch-size group, target position and their interaction) was significantly different from the null model (*χ*^2^ = 17.479, d.f. = 3, *p* < 0.001, *conditional R*^2^ = 0.138; see electronic supplementary material, table S7). We found a significant effect of touch-size group, with individuals of the Touch-Large group being more likely to correctly touch their target than individuals of the Touch-Small group. We also assessed if reaction times differed depending on touch-size group and target position. For correct trials, the full model did not differ significantly from the null model (*χ*^2^ = 5.742, d.f. = 3, *p* = 0.125). For incorrect trials, the full model also did not differ significantly from the null model (*χ*^2^ = 5.047, d.f. = 3, *p* = 0.168).

### 3.2. Different-size test

#### 3.2.1. Descriptive analysis

In the different-size test, we were interested in effects of vertical stimulus position on task performance as a function of target-size group. Table 3 gives an overview of the touch responses for the two possible arrangements; 1315 observations of 11 individuals are included in this dataset (eight trials were excluded due to latencies greater than 3 s).

**Table 3.** Descriptive statistics for different-size test by stimulus arrangement and touch-size group.

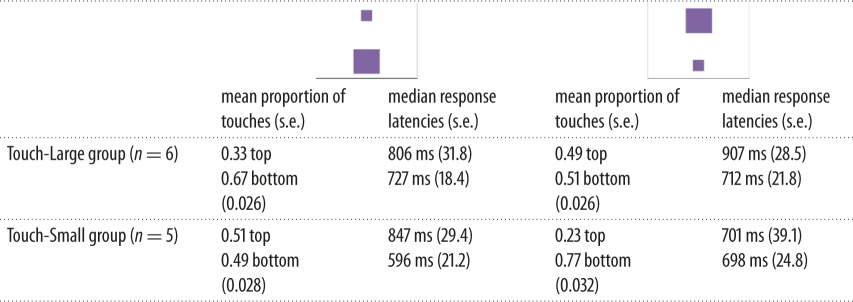

#### 3.2.2 Statistical analysis

##### 3.2.2.1. Accuracy

The full model (containing touch-size group, target position, their interaction and session) was significantly different from the null model (*χ*^2^ = 78.384, d.f. = 4, *p* < 0.001, *conditional R^2^* = 0.071). We found a marginally significant interaction of touch-size group and target location, which corroborates the descriptive result pattern: The monkeys were generally more likely to touch their respective target when it was presented at the bottom location (table 4 for more detailed results). While individuals of both reward groups touched their respective target at chance when it was presented at the top, and both groups were more often correct when their target was presented on the bottom, the Touch-Small group was better than the Touch-Large group when the target was presented at the bottom location. The positive sign of the coefficient for session indicates that the probability to correctly touch the target increased throughout the sessions.

**Table 4.** Results for individual predictors on response accuracy for different-size test (reference categories: Touch-Small group, target position bottom).

term	estimate	s.e.	CI_lower_	CI_upper_	*z*-value	*p*-value
intercept	0.914	0.182	0.561	1.275	5.022	<0.001
Touch-Large group	−0.496	0.177	−0.847	−0.149	−2.801	<0.001
target position top	−1.170	0.179	−1.526	−0.822	−6.522	<0.001
session	0.097					
group × target position	0.424	0.237	−0.039	0.890	1.789	0.074

##### 3.2.2.2. Reaction time

We assessed whether response latencies differed depending on touch-size group and target position, specifically, whether the monkeys would detect their respective target faster when the small stimulus was on the bottom and the large on top (a hierarchy association) or the other way around (an Aristotelian gravitational-weight association). A first assessment of the data revealed a significant difference of response latencies between trials in which the target was touched correctly or not. Subsequently, we analysed the correct and incorrect trials separately. Both models were significantly different from the null model containing only ID as a random factor and both showed increased latencies for touching the upper position (correct trials: *χ*^2^ = 15.311, d.f. = 3, *p* = 0.002, *conditional R^2^* = 0.194; incorrect trials: *χ*^2^ = 14.393, d.f. = 3, *p* = 0.002, *conditional R*^2^ = 0.174). For correct trials, we found that the monkeys were overall faster to touch the target at the lower position, but reaction times did not differ between Touch-Small and Touch-Large group (table 5). For incorrect trials, we found that the monkeys were overall also quicker to wrongly touch the lower position (table 6).

**Table 5.** Results for individual predictors for latency in correct trials different-size test (reference categories: Touch-Large group, target position bottom).

term	estimate	s.e.	CI_lower_	CI_upper_	*χ*^2^	d.f.	*p*-value
intercept	−0.251	0.070	−0.398	0.103	^a^	^a^	^a^
Touch-Small group^b^	−0.021	0.102	−0.239	0.195	3.029	2	0.219
target position top^c^	0.156	0.044	0.069	0.243	14.948	2	<0.001
group × target position^d^	0.095	0.058	−0.208	0.019	2.673	1	0.102

^a^Not shown because of having limited interpretation.

^b^Test was obtained from a likelihood ratio test comparing the full with the reduced model lacking target position.

^c^Test was obtained from a likelihood ratio test comparing the full with the reduced model lacking target touch-size group.

^d^Test was obtained from a likelihood ratio test comparing the full with the reduced model lacking target or their interaction.

**Table 6.** Results for individual predictors for latency in incorrect trials different-size test (reference categories: Touch-Large group, target position bottom).

term	estimate	s.e.	CI_lower_	CI_upper_	*χ*^2^	d.f.	*p*-value
intercept	−0.143	0.068	−0.283	−0.002	^a^	^a^	^a^
Touch-Small group^b^	−0.180	0.098	−0.384	0.021	3.371	2	0.185
target position top^c^	−0.166	0.068	−0.299	−0.033	11.265	2	0.004
group × target position^d^	0.039	0.087	−0.131	0.210	0.205	1	0.651

^a^Not shown because of having limited interpretation.

^b^Test was obtained from a likelihood ratio test comparing the full with the reduced model lacking target position.

^c^Test was obtained from a likelihood ratio test comparing the full with the reduced model lacking target touch-size group.

^d^Test was obtained from a likelihood ratio test comparing the full with the reduced model lacking target or their interaction.

### 3.3. Same-size test

#### 3.3.1. Descriptive analysis

In the same-size test, we were interested in which position the monkeys would touch when they were presented with two vertically arranged identical stimuli, that is, when there was no correct or incorrect response, and if the responses differed as a function of touch-size group. Results of the monkeys’ touch responses are summarized in table 7; 1305 observations of 11 individuals are included in this dataset (12 trials were excluded due to latencies greater than 3 s).

**Table 7.** Descriptive statistics for same-size test.

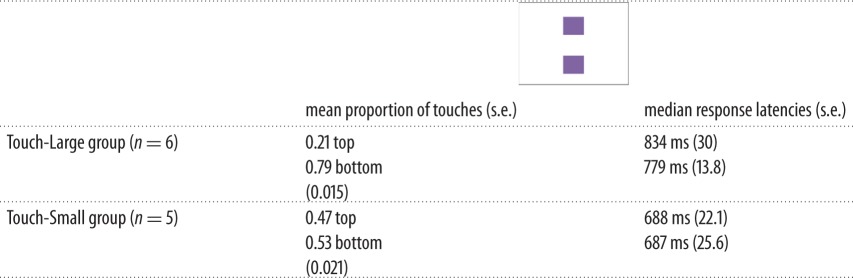

#### 3.3.2. Statistical analysis

##### 3.3.2.1. Touch position

The full model (containing touch-size group and session) was significantly different from the null model (*χ*^2^ = 51.416, d.f. = 2, *p* < 0.001, *conditional R^2^* = 0.424) (full model results in table 8). We found a significant effect of touch-size group on the probability to touch the bottom position, with the individuals of the Touch-Large group touching the stimulus on the bottom position more often than individuals of the Touch-Small group. The positive coefficient of ‘session’ indicates an increasing tendency to touch the stimulus on the lower position.

**Table 8.** Results for individual predictors on probability to touch the bottom position in the same-size test (reference category: Touch-Small group).

term	estimate	s.e.	CI_lower_	CI_upper_	*z*-value	*p*-value
intercept	−0.847	0.551	−2.051	0.349	−1.539	0.124
Touch-Large group	1.841	0.735	0.292	3.522	2.503	0.012
session	0.320					

##### 3.3.2.2. Reaction time

We also assessed if reaction times differed depending on touch-size group. This was not the case, as the full model containing this variable of interest did not have higher explanatory value than the null model with random factor ID only (*χ*^2^ = 0.605, d.f. = 1, *p* = 0.437).

## 4. Discussion and Study 2

The main findings of Study 1 are the following: First, it was easier for the monkeys to learn to touch the larger of two stimuli and to generalize this rule to new shapes in the horizontal training (see electronic supplementary material for more details on the horizontal training). Second, in the different-size test, both groups had a tendency to touch their respective target stimulus more often correctly when it was presented at the bottom compared to the top of the screen. Third, both groups experienced a drop in performance when stimuli were presented in vertical arrangement. Fourth, despite their difficulties in the horizontal training, the Touch-Small group outperformed the Touch-Large group in the different-size test (more correct responses). Finally, in the same-size test, the Touch-Large group had a pronounced bias to touch the stimulus on the bottom position while the Touch-Small group had no overall preference for any position.

Taken together, this pattern does not support either hypothesis. The observation that the subjects in the Touch-Large group chose the bottom position more often than the top position (same-size test) supports a naive Aristotelian weight representation of size and verticality. However, their performance in the different-size test does not, because they should have been even more accurate than 67% when their target (which was supposedly even the easier of the two target sizes) was presented at the preferred bottom position. In general, the Touch-Large group's sharp drop in accuracy performance in the different-size test is surprising considering their faster learning of the task during the horizontal training.

We suggest that the performance of the Touch-Large group conformed with an innate bias to choose the larger stimulus, while the Touch-Small group had to suppress that bias and employ greater executive control. To assess the influence of task proficiency on performance in the two groups, we ran an explorative follow-up study with a subset of five individuals of the original study, in which we trained individuals of both groups to the same level of proficiency in the different-size task. We were interested in knowing if the group difference in bias to touch the bottom position when two identical stimuli are presented would replicate, or whether the bias to touch the bottom position would disappear in the Touch-Large group once they are better at the vertical different-size task. As in Study 1, Touch-Large group individuals expressed a stronger tendency to touch the lower of two identical medium-sized stimuli in the same-size test (proportions of touching the bottom position: *m*_large_ = 77%, *m*_small_ = 57%). We found no statistical evidence for this difference (*χ*^2^ = 1.369, d.f. = 1, *p* = 0.242); however, our sample size was very small and our data might have lacked the power to replicate the effects of Study 1. A detailed description of the procedure and results are provided in the electronic supplementary material. In the light of the response pattern of Study 2, we find it unlikely that the group difference in the same-size test of Study 1 can be attributed to differences in the groups' understanding of the task.

## 5. General discussion

We explored whether long-tailed macaques’ response to a larger or smaller stimulus was influenced by vertical spatial position of the stimulus on a touch screen. We contrasted two hypotheses: on the one hand, monkeys might display similar sociations as humans, in whom large is associated with top and small is associated with bottom [28]. Alternatively, monkeys might employ an Aristotelian association of large with bottom and small with top, following the gravitational rationale that larger objects of the same kind are heavier and thus strive more to the ground.

In contrast to predictions of either hypothesis, both Touch-Large and Touch-Small individuals had a tendency to touch their respective target stimulus more when it was presented at the bottom of the screen. This effect was even more pronounced for the Touch-Small group in comparison to the Touch-Large group. When the respective target stimulus was presented at the top of the screen, both groups performed at chance level. This might indicate that they were guessing randomly, or it could be the product of two conflicting strategies, namely the bias to touch the bottom position, and the aim to touch the correct stimulus. Interestingly, individuals of both groups also responded faster when touching the bottom position irrespective of whether this was their target stimulus or not; and latencies of incorrect touches were overall shorter than of correct touches. It is unlikely that this pattern (preference for and shorter response latencies at the bottom position) is merely a consequence of the physical distance and convenience to reach the stimulus at the bottom: first, we observed no systematic differences between individuals of different body size regarding a preferred position to touch and second, it is also important to note that our monkeys were not physically restricted during testing and could change their body position (sitting, standing, turning, even climbing) as they preferred. In the same-size test, Touch-Large individuals expressed a clear and increasing bias to touch the bottom position. Touch-Small individuals, on the other hand, overall touched both positions similarly often. This renders it unlikely that the bottom position had a stronger general association with being rewarded, because in this case, we would also expect the Touch-Small group to preferentially touch the bottom position (they were more accurate at that position in the different-size test overall). Taken together, our monkeys did not express a clear response pattern that favours either the hierarchy or weight size-verticality association. Hence, at first sight, our results do not support the idea that size comparisons are grounded in vertical location. Instead, our data indicate a spontaneous bias of the monkeys to choose larger-sized stimuli and to touch stimuli at the lower position. Our results most likely emerged from a joint influence of both these biases, because neither of the two processes can account for the difference between touch-size groups in the *same-size test* as well as the lack of difference between trials with different stimulus arrangements in the *different-size test*.

It is important to note that we cannot exclude the possibility that the monkeys learned an absolute size rule rather than to touch the relatively smaller or larger stimulus. This would not explain the difference between the small and the large group, however. It could further be argued that the poor transfer from horizontal to vertical condition is a consequence of the monkeys having acquired a very specific set of individual rules for each shape and horizontal position, which enabled them to successfully pass the horizontal training but did not work in the vertical arrangement. Given their generalization training and performance in the horizontal training phase, we think this is unlikely. It would also not explain the group difference (i.e. why would small group individuals be less affected than large group individuals?).

We also found that individuals of the two different touch-size groups learned and generalized the tasks differently during the horizontal training. Specifically, it was easier for the monkeys to learn to touch the larger of two stimuli, and the transition from two to six stimuli seemed to be harder for the Touch-Small group. While all subjects in the Touch-Large group immediately reached the criterion in the last horizontal training stage, none of the individuals in the Touch-Small group showed a similar ceiling effect; two of these individuals in fact needed two to three times longer to reach the criterion. We had not expected that the difference between the two groups would be as pronounced, although several studies had reported a preference for larger food quantities and accompanying difficulties to solve reverse-contingency tasks (where the individual has to choose the smaller quantity in order to receive the larger quantity) (see [41] for the role of item size and quantity on choice behaviour in chimpanzees).

In our study, the monkeys of both groups were less accurate when the task switched from horizontal to vertical arrangement—even the individuals in the Touch-Large group, who had immediately generalized to the novel shapes, were much less accurate than in the horizontal training. Interestingly, the subjects in the Touch-Large group experienced a sharper drop in performance than the subjects in the Touch-Small group. This is surprising given their excellent performance and fast learning of the task in the horizontal training. One possibility to explain this pattern is that the Touch-Small group needed to exert greater executive control because they had to suppress the innate disposition to touch the larger stimulus. This means that, from the beginning, touching the small stimulus presented a more difficult task than touching the large stimulus. This would explain both the relatively prolonged training phase of the Touch-Small group compared to the Touch-Large group and the better performance of the Touch-Small group in the vertical set-up. In the different-size test, all monkeys had a bias to touch the bottom position (as visible in both groups' above-chance accuracy performance at the bottom position), but as a consequence of the higher cognitive load for the Touch-Small group, these individuals were more concentrated during the task and touched their target stimulus more often correctly than individuals of the Touch-Large group. In the same-size test, the influence of both predispositions (touch larger stimulus and touch stimulus at the bottom) added up and resulted in the strong bias to touch the bottom position for the Touch-Large group. The Touch-Small group, on the other hand, had learned to suppress their predisposition and to focus harder on selecting according to size, which resulted in touching both positions at roughly equal rates. This explanation suggests that a combination of biases as well as executive control demands contribute to the observed performance patterns. One might argue that, usually, increased executive control is associated with longer response latencies and we admit that this was not the case in our monkeys. On the other hand, however, the monkeys had no specific motivation to react as fast as possible, i.e. there was neither a time-out for responding nor were they food or water deprived and thus in need of performing fast. It is equally possible that different processes (such as increased executive control requirements for the Touch-Small group and distractibility as a consequence of reduced focus on the task with little executive control requirement for the Touch-Large group) resulted in similar response latencies.

In summary, the results of this study do not provide conclusive evidence for the question if and in which direction stimulus size is mentally associated with vertical position in monkeys. We have discussed the possibility that the presently found pattern of performance emerges from a joint influence of biases towards large objects and bottom positions and executive control demands. Further investigation of magnitude-space mappings in behavioural studies both with human children of different age and non-human primates might help disentangle ontogenetic and phylogenetic differences and clarify the boundaries of spatial-numerical associations (regarding both for which type of magnitude information as well as to which spatial dimension these common associations apply). Future research using neuroimaging techniques could focus on whether spatial-numerical mapping on the vertical dimension is not as intimately wired on shared neuronal representations as on the horizontal dimension (for findings indicating a close connection between spatial and numerical magnitude processing and a crucial role of parietal associative cortical regions, in which these processes share some neuronal circuitry, see for example [2,12–14]) and whether similar cortical regions are engaged in processing of spatial information and abstract concepts (such as relating social status with verticality). Finally, the response format might play a role in whether spontaneous magnitude-spatial associations occur or not; so far different studies have used different tasks to identify mapping processes (e.g. match to sample, two-choice discrimination, spatial cueing and looking behaviour), which makes it hard to disentangle effects of response format, study group and type of stimuli.

## Supplementary Material

ESM - Details on stimuli and results

## Supplementary Material

ESM - Data

## Data accessibility

The dataset supporting this article has been uploaded as part of the electronic supplementary material.
