# Supplementary material for: Are monkeys intuitive Aristotelians? Associations between target size and vertical target position in long-tailed macaques
Source: R Soc Open Sci. 2018 Apr 11;5(4):170889. doi: 10.1098/rsos.170889 (PMC5936887; doi:10.1098/rsos.170889)
Supplement: ESM - Details on stimuli and results [file rsos170889supp1.pdf]

Stimuli

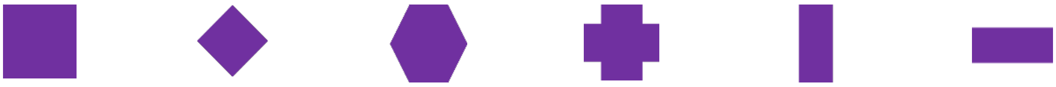

Square | Diamond | Hexagon | Cross | Vertical rectangle | Horizontal rectangle

Figure S1 Experimental stimuli

Study 1

Additional results for horizontal training stages

Stage 1 (square only)

Table S1

Descriptive statistics for horizontal training stage 1 by stimulus arrangement and touch-size group.

	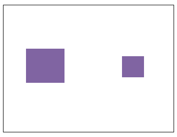		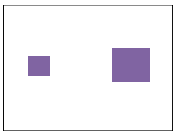	
	Mean proportion of touches (*SE*)	Median response latencies (*SE*)	Mean proportion of touches (*SE*)	Median response latencies (*SE*)
Touch-Large group (n=6)	0.15 Right<br>0.85 Left<br>(.021)	718 ms (57.1)<br>657 ms (29.2)	0.37 Right<br>0.63 Left<br>(.029)	837 ms (41.9)<br>575 ms (28.4)
Touch-Small group (n=5)	0.5 Right<br>0.5 Left<br>(.026)	609 ms (22.9)<br>631 ms (25.3)	0.5 Right<br>0.5 Left<br>(.027)	580 ms (17)<br>605 ms (26.3)

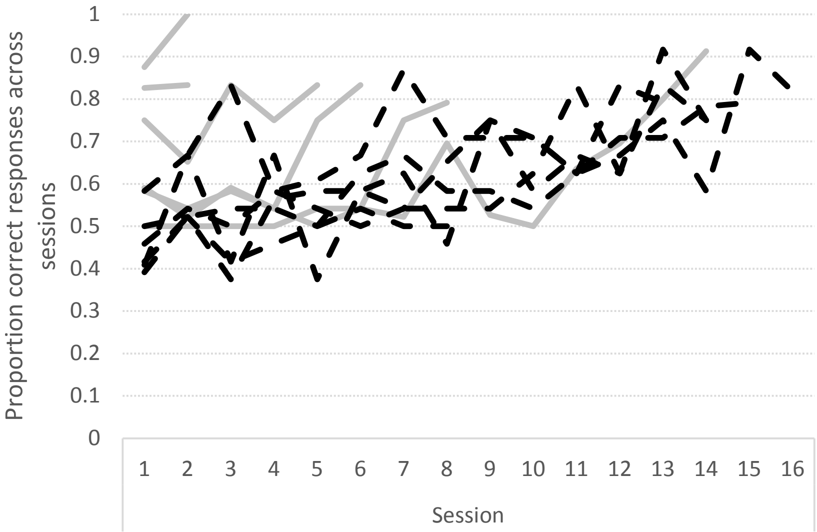

Figure S2 Individual performances during horizontal training stage 1. Grey solid lines=Touch-Large group individuals, black dashed lines=Touch-Small group individuals.

Table S2

Results for horizontal training stage 1: individual predictors for response accuracy (reference categories: Touch-Small group, Target position left)

Term	Estimate	SE	CI_{lower}	CI_{upper}	z-value	p-value
Intercept	0.018	0.313	-0.664	0.699	0.06	0.955
Touch-Large group	2.252	0.476	1.293	3.322	160.51	<.001
Target position Right	-0.011	0.151	0.307	0.285	100.52	.941
Group×Target position	-2.520	0.271	-3.061	-1.996	96.460	<.001

Table S3

Results for horizontal training stage 1: individual predictors for latency in correct trials (reference categories: Touch-Large group, Target position left)

Term	Estimate	SE	CI_{lower}	CI_{upper}	χ^2	df	p
Intercept	-0.324	0.093	-0.519	-0.126	(1)	(1)	(1)
Touch-Small group(2)	-0.228	0.133	-0.511	0.052	2.723	2	.256
Target position Right(3)	0.103	0.052	0.001	0.205	7.527	2	.023
Group×Target position(4)	0.006	0.077	-0.146	0.158	0.006	1	.939

(1)) Not shown because of having limited interpretation

(2-4) indicated test was obtained from a likelihood ratio test comparing the full with the reduced model lacking target position(2), touch-size group(3), or their interaction(4)

Stage 2 (two shapes)

Table S4

Descriptive statistics for horizontal training stage 2 by stimulus arrangement and touch-size group.

	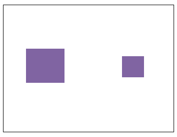		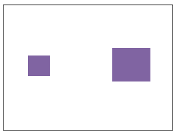	
	Mean proportion of touches (*SE*)	Median response latencies (*SE*)	Mean proportion of touches (*SE*)	Median response latencies (*SE*)
Touch-Large group (n=6)	0.24 Right<br>0.76 Left<br>(.023)	689 ms (47.2)<br>705 ms (25.3)	0.86 Right<br>0.14 Left<br>(.018)	702 ms (19.9)<br>623 ms (41.6)
Touch-Small group (n=5)	0.6 Right<br>0.4 Left<br>(.028)	696 ms (20.5)<br>583 ms (40.2)	0.33 Right<br>0.67 Left<br>(.027)	584 ms (31.6)<br>632 ms (25)

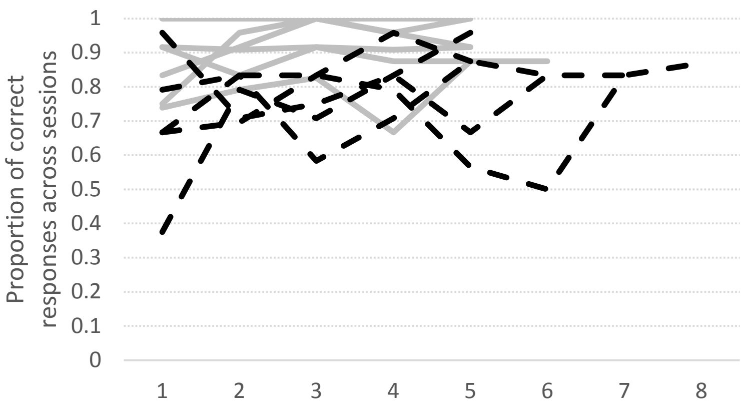

Figure S3 Individual performances during horizontal training stage 2. Grey solid lines=Touch-Large group individuals, black dashed lines=Touch-Small group individuals.

Table S5

Results for horizontal training stage 2: individual predictors for response accuracy (reference categories: Touch-Small group, Target position left)

Term	Estimate	SE	CI_{lower}	CI_{upper}	z-value	p-value
Intercept	0.696	0.203	0.257	1.138	3.421	.001
Touch-Large group	0.534	0.283	-0.055	1.163	1.886	.059
Target position Right	-0.277	0.171	-0.614	0.058	-1.620	.105
Group×Target position	0.957	0.263	0.443	1.477	3.637	<0.001

Stage 3 (all shapes)

Table S6

Descriptive statistics for horizontal training stage 3 by stimulus arrangement and touch-size group.

	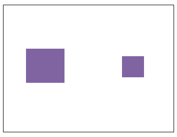		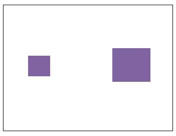	
	Mean proportion of touches (*SE*)	Median response latencies (*SE*)	Mean proportion of touches (*SE*)	Median response latencies (*SE*)
Touch-Large group (n=6)	0.15 Right<br>0. 85 Left<br>(.019)	662 ms (73.3)<br>717 ms (26.9)	0.86 Right<br>0.14 Left<br>(.018)	787 ms (21.9)<br>596 ms (39.5)
Touch-Small group (n=5)	0.59 Right<br>0.41 Left<br>(.029)	606 ms (22.8)<br>477 ms (27.3)	0.35 Right<br>0.65 Left	511 ms (20.9)<br>587 ms (17.3)

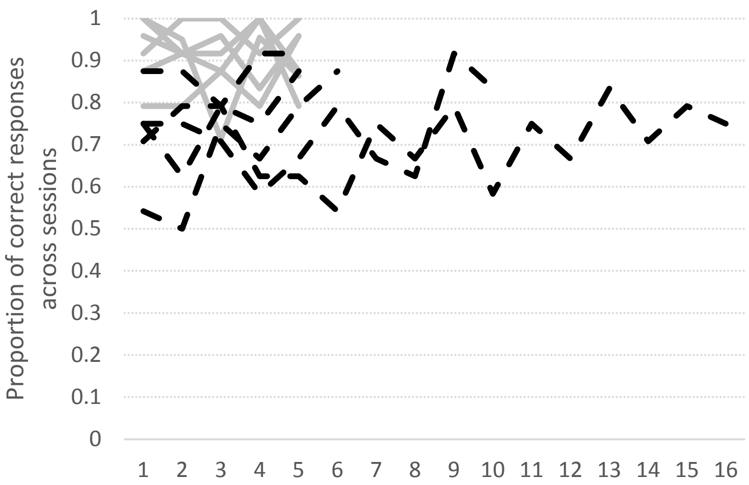

Figure S4 Individual performances during horizontal training stage 3. Grey solid lines=Touch-Large group individuals, black dashed lines=Touch-Small group individuals.

Table S7

Results for horizontal training stage 3: individual predictors for response accuracy (reference categories: Touch-Small group, Target position left)

Term	Estimate	SE	CI_{lower}	CI_{upper}	z-value	p-value
Intercept	0.645	0.186	0.255	1.041	3.475	.001
Touch-Large group	1.121	0.272	0.569	1.702	4.120	<.001
Target position Right	-0.266	0.170	-0.600	0.067	-1.563	.118
Group×Target position	0.355	0.274	-0.183	0.894	1.295	.195

Study 2

Methods and Results

Subjects

Based on availability, a subset of individuals (n = 5) from Study 1 was chosen to participate in the follow-up Study 2. Specifically, two individuals of the large group (Mars, Milka) and three individuals of the small group (Mila, Snickers, Selina) were tested.

Procedure

All individuals were trained in the different-size task until they performed reliably above chance, specifically until they correctly touched their target stimulus at least at 91.7 % (i.e., ≥ 22 correct out of 24 trials) in two consecutive sessions. The same stimuli and reward scheme was used as in Study 1. Once an individual reached this criterion, they received five sessions of the same-size test.

Results

Vertical training. The two individuals of the large group reached the new training criterion after 11 and 19 sessions respectively. One of them performed above the training criterion from Study

1 (75 % correct in two consecutive sessions) from the beginning and the second individual reached it after 6 sessions. The three individuals of the Touch-Small group reached the new criterion after 20, 23 and 27 sessions respectively. They reached the old Study 1 criterion after 15 and 16 sessions.

Same-size test.

Descriptive analysis. In the same-size test, we were interested which position the monkeys would touch when they were presented with two vertically arranged identical stimuli (similar to Study 1, when there was no correct or incorrect answer). Results of the monkeys' touch responses are summarized in table S7; 599 observations of 5 individuals are included in this dataset. As in Study 1, the subjects in the Touch-Large group had a greater tendency to touch the bottom position than subjects in the Touch-Small group.

Table S8
Descriptive statistics for same-size test Study 2

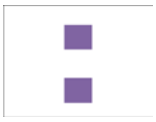

	Mean proportion of touches (*SE*)	Median response latencies (*SE*)
Touch-Large group (n=2)	0.33 Top<br>0.77 Bottom<br>(.027)	834 ms (25.8)<br>664 ms (22.4)
Touch-Small group (n=3)	0.43 Top<br>0.57 Bottom<br>(.026)	688 ms (15)<br>575 ms (20.8)

Statistical analysis. The full model (containing reward category and session) was not different from the null model (χ^2= 1.369, *df*=1, *p*= .242), i.e., the group difference in likelihood to touch the stimulus at the bottom position (Touch-Large group: 77 %, Touch-Small group: 57 %) did not reach statistical significance. We also assessed if reaction times differed depending on reward category and touch position. The full model containing reward category, touch position and their interaction (see table S9), was significantly different from the null model with random factor ID only (χ^2=59.854, *df*=3, *p*<.001). We found that in general, the monkeys were faster in touching the stimulus at the bottom.

Table S9

Results for individual predictors on response latency for same-size test in Study 2 (reference categories: Touch-Small group, Target position bottom)

Term	Estimate	SE	CI_{lower}	CI_{upper}	χ^2	df	p
Intercept	-0.536	0.066	-0.695	-0.380			
Touch-Small group[(2)]	0.193	0.104	-0.054	0.443	2.725	2	.256
Target position top[(3)]	0.210	0.032	0.146	0.273	57.008	2	<.001
Group×Target position[(4)]	-0.021	0.054	-0.127	0.086	0.148	1	.700

[(1)] Not shown because of having limited interpretation

[(2-4)] indicated test was obtained from a likelihood ratio test comparing the full with the reduced model lacking target position[(2)], touch-size group[(3)], or their interaction[(4)]
